# Supplementary material for: Integrator is recruited to promoter‐proximally paused RNA Pol II to generate Caenorhabditis elegans piRNA precursors
Source: EMBO J. 2020 Dec 19;40(5):e105564. doi: 10.15252/embj.2020105564 (PMC7917550; doi:10.15252/embj.2020105564)
Supplement: Supplementary file 1 — Appendix [file EMBJ-40-e105564-s001.pdf]

Appendix

Table of contents

1. **Appendix Supplementary Figures** 2

a. Appendix Fig. S1: Integrator localizes to sites of piRNA biogenesis in germ cells 2
b. Appendix Fig. S2: piRNA precursor length distribution of individual replicates 4
c. Appendix Fig. S3: Further analysis of cleavage fragment abundance 6
d. Appendix Fig. S4: Integrator and the strength of AT-rich termination signals 8
e. Appendix Fig. S5: Comparison of nucleoplasmic and chromatin-bound piRNA precursor length distributions 9
f. Appendix Fig. S6: Transcriptional changes in snRNAs and protein-coding genes upon *ints*-11 knockdown 10
g. Appendix Fig. S7: Transcriptional changes in transposable elements upon ints-11 knockdown 12
h. Appendix Fig. S8: Overview of cloning methods used in this work 13

2. **Appendix Supplementary Tables** 14

a. Appendix Table S1: C. elegans strains 14
b. Appendix Table S2: Oligonucleotides 14

1. Appendix Supplementary Figures

A

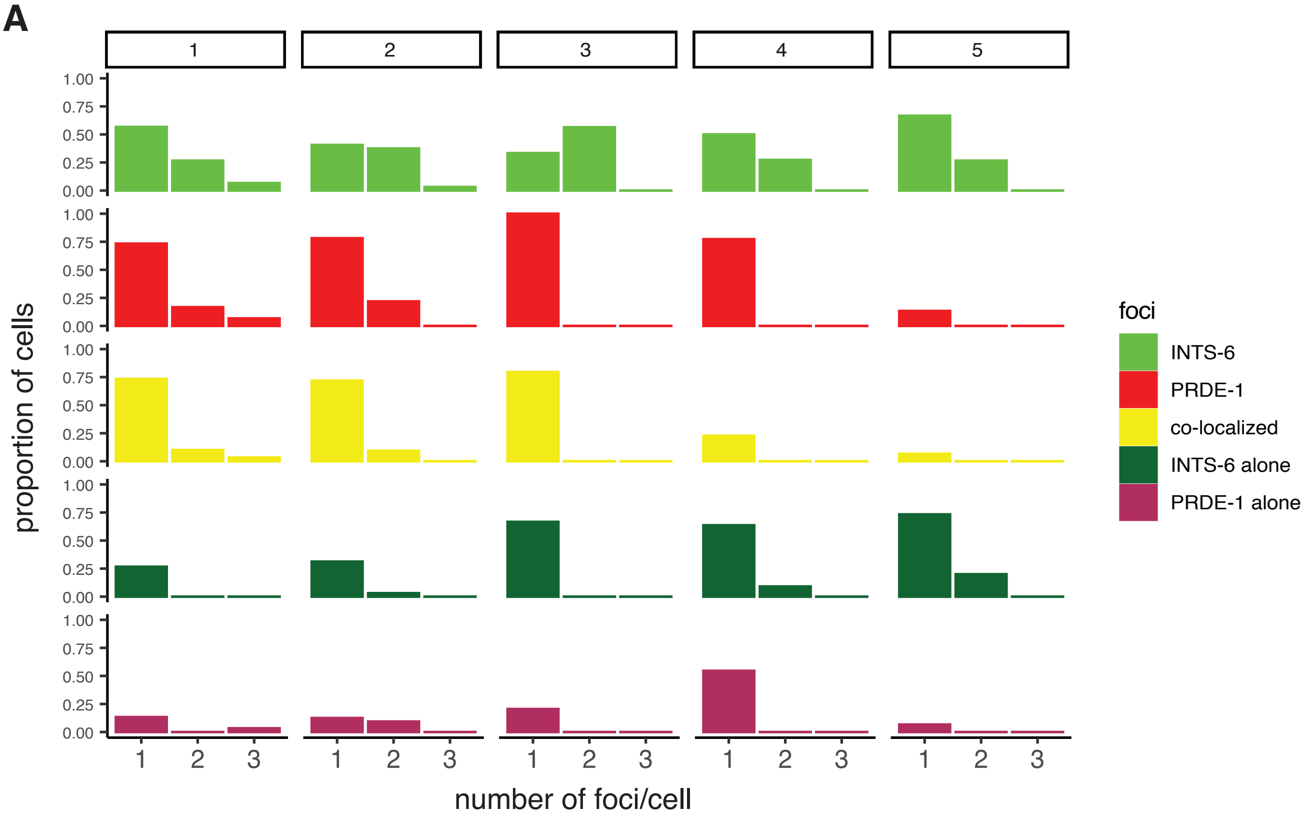

B

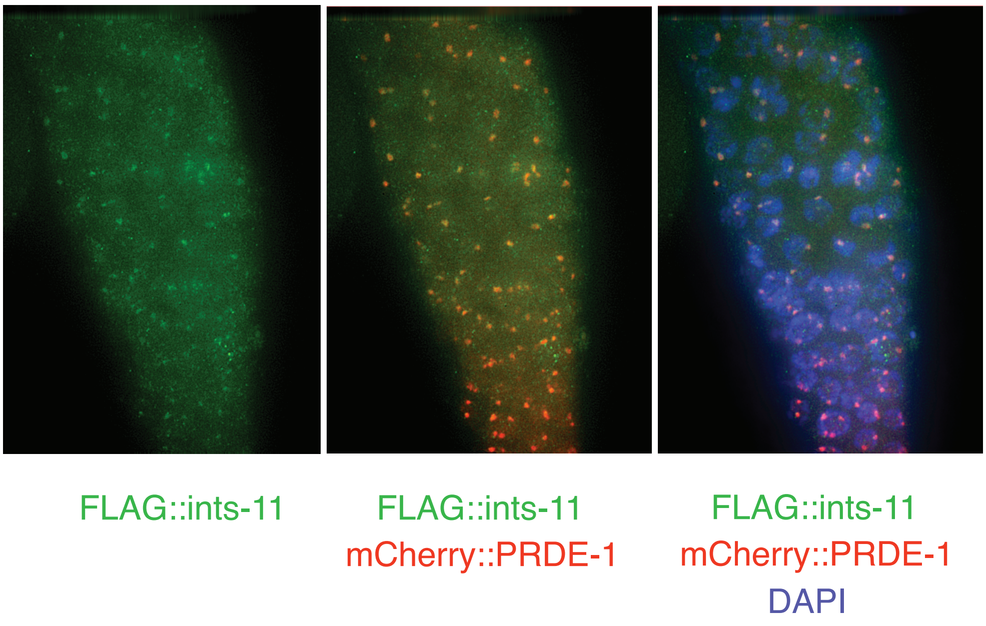

Appendix Figure S1. Integrator localizes to sites of piRNA biogenesis in germ cells (relates to Figure 2).

A. Quantification of the number of INTS-6::GFP and mCherry::PRDE-1 foci/cell and their co-localization across the *C. elegans* germline. 1-5 correspond to the germline sections shown in Figure 2B.
B. Co-localization of FLAG::INTS-11 and mCherry::PRDE-1 in the *C. elegans* germline observed by anti-FLAG and anti-mCherry immunofluorescent staining.

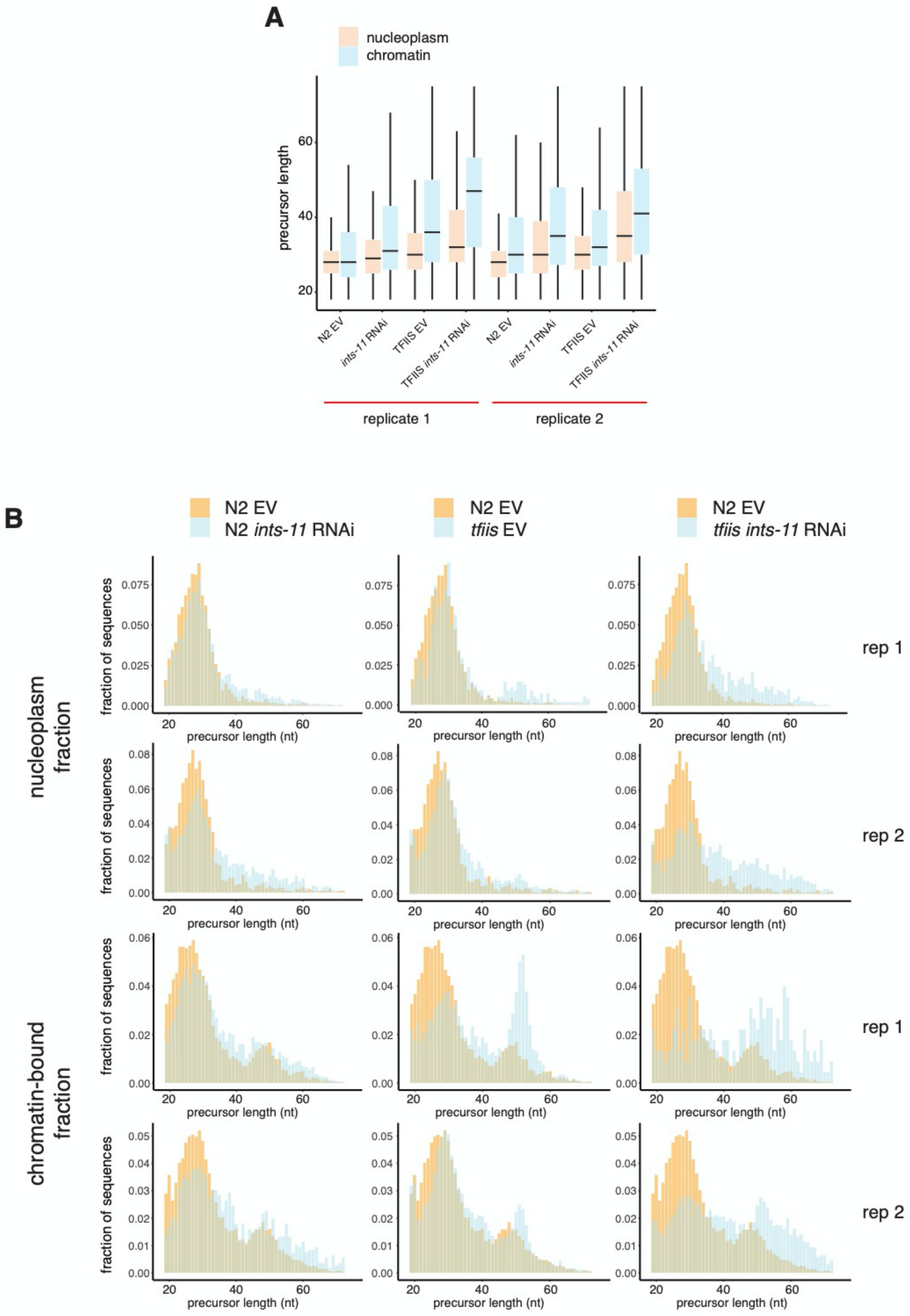
A
nucleoplasm
chromatin
precursor length
60
40
20
N2 EV
ints-11 RNAi
TFIIS EV
TFIIS ints-11 RNAi
replicate 1
replicate 2
B
N2 EV
N2 ints-11 RNAi
tfiis EV
tfiis ints-11 RNAi
nucleoplasm fraction
chromatin-bound fraction
fraction of sequences
precursor length (nt)
rep 1
rep 2

Appendix Figure S2. piRNA precursor length distribution of individual replicates (relates to Figure 3).

A. Boxplots comparing the length of pairs of nucleoplasmic and chromatin-libraries corresponding to the same initial nuclei sample.

B. Length distribution of chromatin-bound and nucleoplasmic piRNA precursors in the two individual replicates of corresponding to each condition.

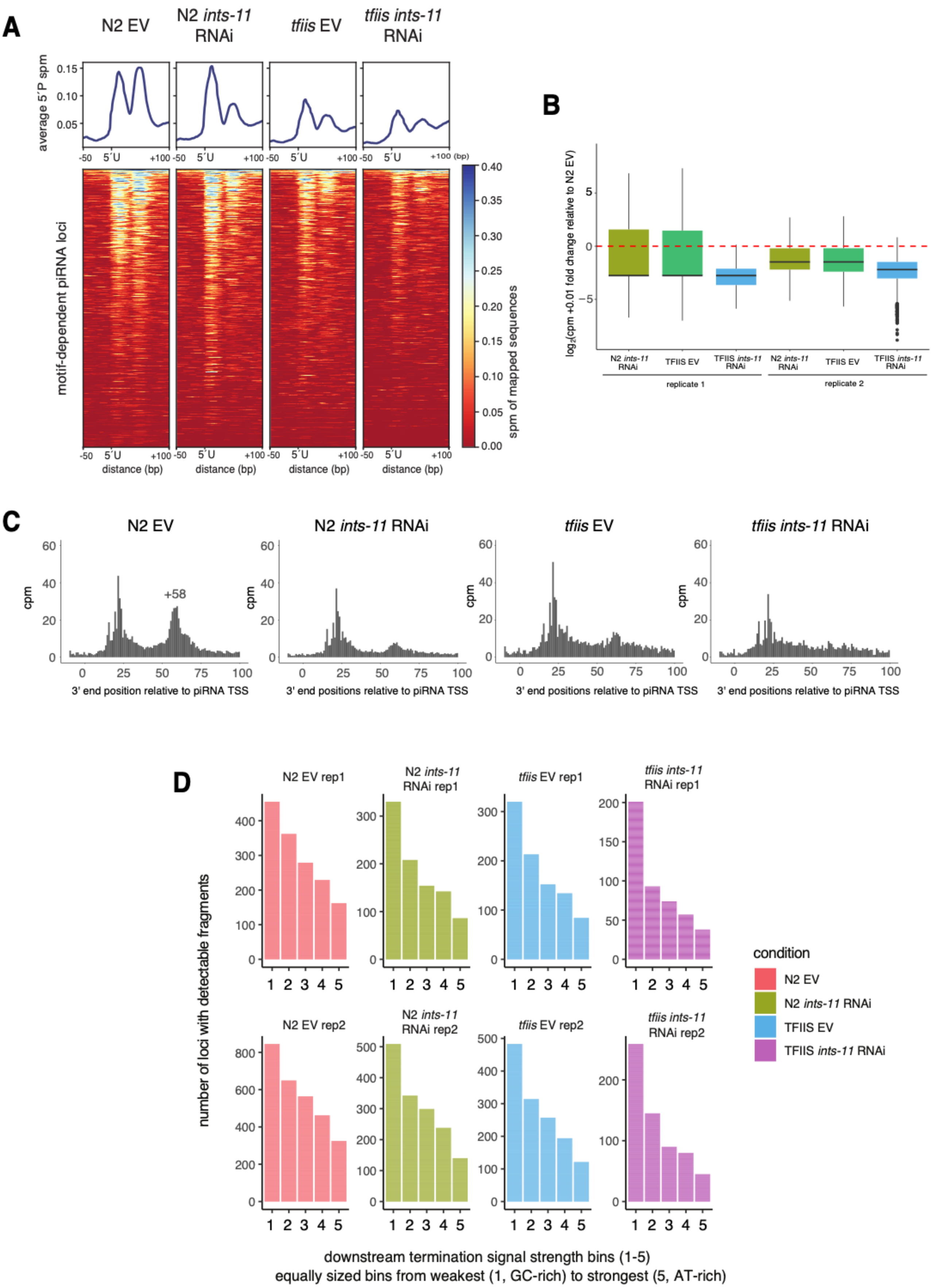
A
N2 EV
N2 ints-11 RNAi
tfiis EV
tfiis ints-11 RNAi
average 5´P spm
0.15
0.10
0.05
-50
5´U
+100
(bp)
motif-dependent piRNA loci
distance (bp)
spm of mapped sequences
0.40
0.35
0.30
0.25
0.20
0.15
0.10
0.05
0.00
B
log2(cpm +0.01 fold change relative to N2 EV)
5
0
−5
N2 ints-11 RNAi
TFIIS EV
TFIIS ints-11 RNAi
replicate 1
replicate 2
C
N2 EV
N2 ints-11 RNAi
tfiis EV
tfiis ints-11 RNAi
cpm
60
40
20
0
+58
0
25
50
75
100
3' end position relative to piRNA TSS
3' end positions relative to piRNA TSS
D
N2 EV rep1
N2 ints-11 RNAi rep1
tfiis EV rep1
tfiis ints-11 RNAi rep1
N2 EV rep2
N2 ints-11 RNAi rep2
tfiis EV rep2
tfiis ints-11 RNAi rep2
number of loci with detectable fragments
1 2 3 4 5
condition
N2 EV
N2 ints-11 RNAi
TFIIS EV
TFIIS ints-11 RNAi
downstream termination signal strength bins (1-5)
equally sized bins from weakest (1, GC-rich) to strongest (5, AT-rich)

Appendix Figure S3. Further analysis of cleavage fragment abundance (relates to Figure 4).

A. Heatmap showing the coverage of unique sequences mapping to piRNA loci after removal of reads corresponding to mature 21U-RNAs (see Methods). The first peak corresponds to 21U-RNA degradation products and 5′ PPP initiation products starting at piRNA TSSs. The second peak corresponds to degradation fragments initiating at +38 on average (Figure 4D), resulting in sequence coverage peaking at +48 due to a median length of fragments of 20 nt (Figure 4D).
B. Log_2 fold change distributions of degradation fragment counts per million of mapped reads relative to N2 EV. Fold changes were calculated after addition of 0.01 pseudocpms. Two independent biological replicates are shown.
C. Positions of 3′ ends of 5′ P fragments relative to piRNA TSSs. Signal is normalized to counts per million mapped reads.
D. Number of loci with detectable cleavage fragments across bins of loci stratified according to the strength of their termination signals, from weak (GC-rich) to strong (AT-rich). Data from each individual library is shown.

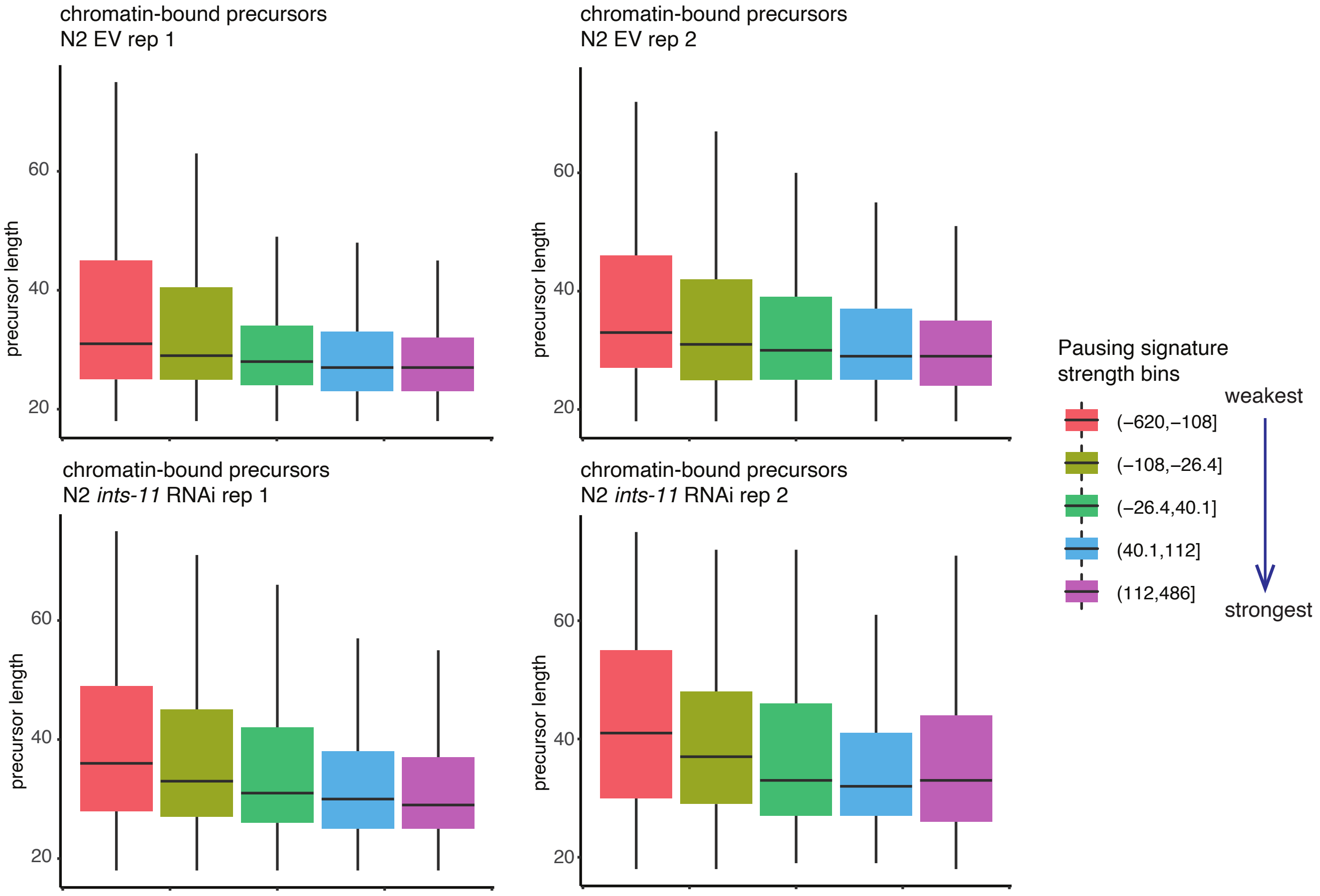

Appendix Figure S4. Integrator and the strength of AT-rich termination signals (relates to Figure 4).
Length distribution of chromatin-bound motif-dependent piRNA precursors stratified by the strength of downstream termination signals from weak (GC-rich) to strong (AT-rich), in empty vector and *ints*-11 RNAi-treated nematodes. Two replicates for each condition are shown.

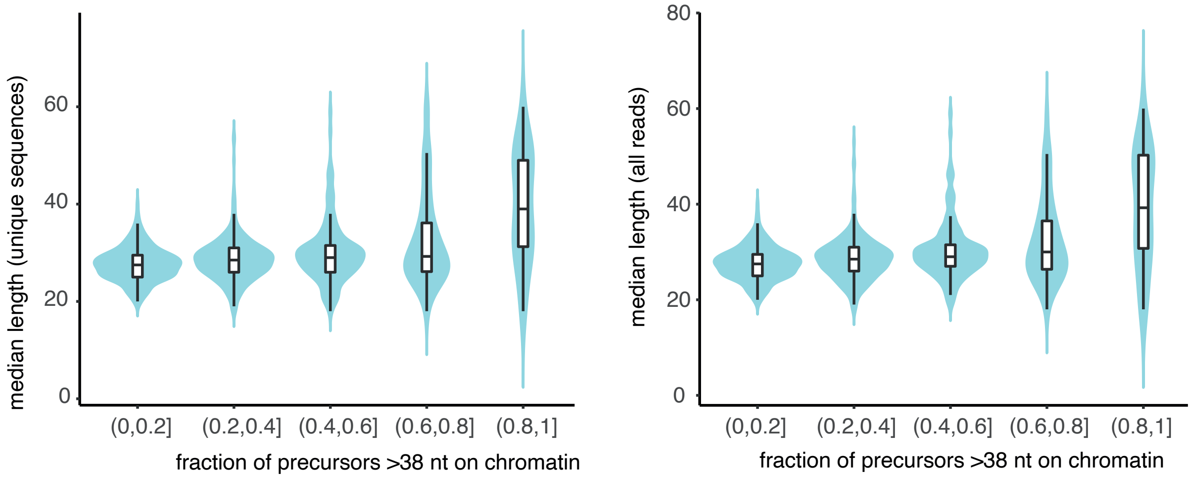

Appendix Figure S5. Comparison of nucleoplasmic and chromatin-bound piRNA precursor length distributions.

Length distributions of nucleoplasmic piRNA precursors in bins of loci with increasing fractions of long (>38 nt) chromatin-bound piRNA precursors. The panel in the left shows the distributions of per-locus median lengths for all unique sequences observed at each locus, while the right panel shows the distributions of per-locus median lengths derived from all reads observed at each locus.

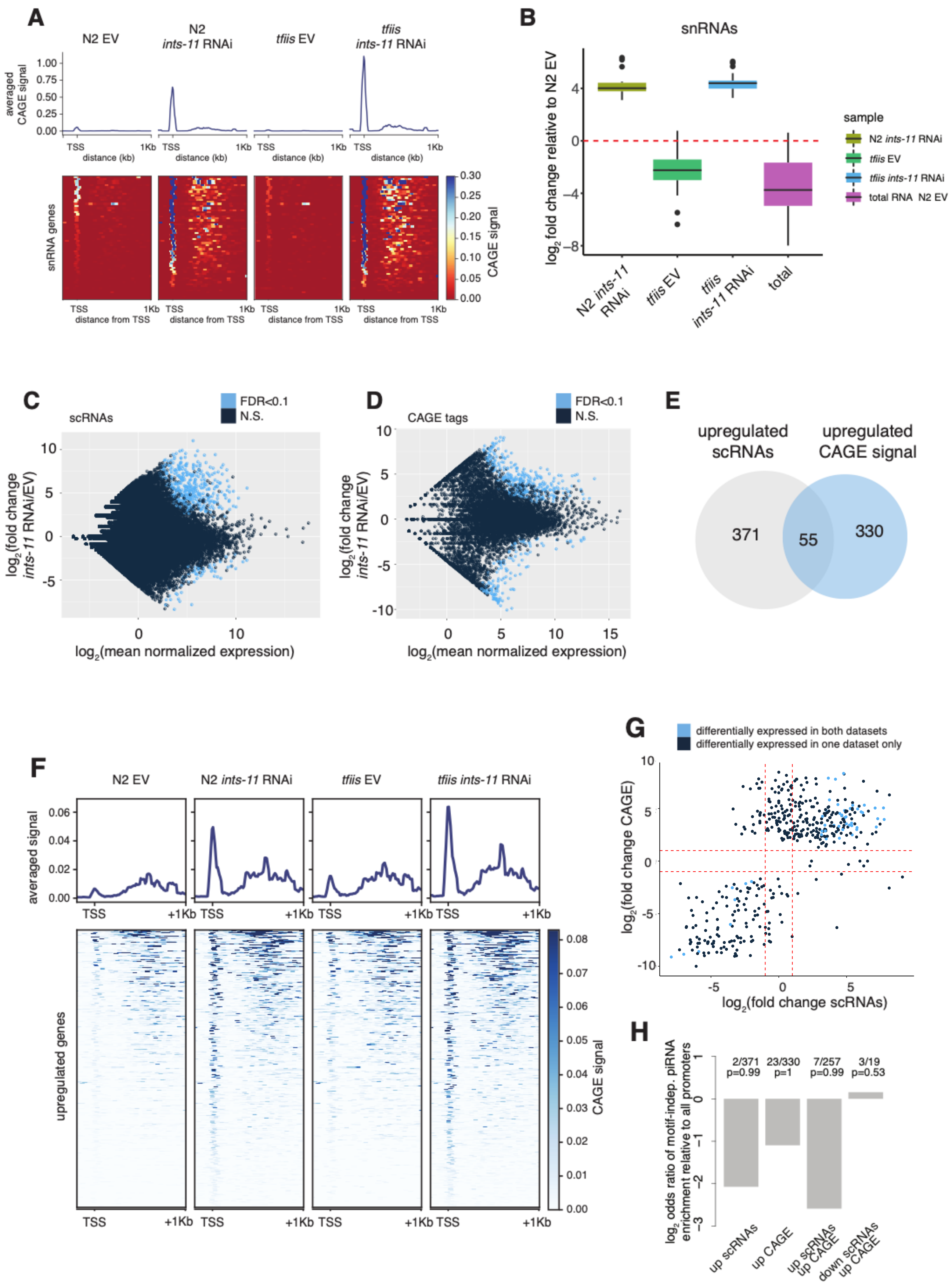
A
N2 EV
N2 ints-11 RNAi
tfiis EV
tfiis ints-11 RNAi
averaged CAGE signal
distance (kb)
snRNA genes
distance from TSS
CAGE signal
B
snRNAs
log2 fold change relative to N2 EV
sample
N2 ints-11 RNAi
tfiis EV
tfiis ints-11 RNAi
total RNA N2 EV
C
scRNAs
FDR<0.1
N.S.
log2(fold change ints-11 RNAi/EV)
log2(mean normalized expression)
D
CAGE tags
E
upregulated scRNAs
upregulated CAGE signal
371
55
330
F
averaged signal
upregulated genes
CAGE signal
G
differentially expressed in both datasets
differentially expressed in one dataset only
log2(fold change CAGE)
log2(fold change scRNAs)
H
log2 odds ratio of motif-indep. piRNA enrichment relative to all promoters
2/371 p=0.99
23/330 p=1
7/257 p=0.99
3/19 p=0.53
up scRNAs
up CAGE
up scRNAs up CAGE
down scRNAs up CAGE

Appendix Figure S6. Transcriptional changes in snRNAs and protein-coding genes upon *ints*-11 knockdown (relates to Figure 5).
A. Nascent CAGE signal at snRNA genes upon *ints*-11 knockdown, in a wild-type and a *tfiis* mutant background. Extensive readthrough transcription with ~500nt fragments is readily detectable upon *ints*-11 knockdown.
B. Distributions of log_2 fold changes in nascent CAGE signal at snRNA loci upon *ints*-11 knockdown, in a wild-type and a *tfiis* mutant background, using N2 empty vector as a baseline.
C. MAplot showing differentially expressed protein-coding genes at the nascent short capped RNA level, with an excess of upregulated genes.
D. MAplot showing differentially expressed protein-coding genes detected by nascent CAGE, with an excess of upregulated genes.
E. Overlap between upregulated genes in the nascent scRNA and CAGE datasets.
F. Nascent CAGE signal at the set of upregulated protein coding genes upon *ints*-11 knockdown, in a wild-type and a *tfiis* mutant background.
G. Correlation between changes in nascent short-capped RNA and CAGE signal, for genes detected as differentially expressed in at least one of the two assays.
H. Log_2 odds ratios of enrichment of motif-independent piRNA loci in protein-coding gene promoters detected as differentially expressed upon *ints*-11 knockdown, at the nascent scRNA level, at the CAGE level, or both (see labels).

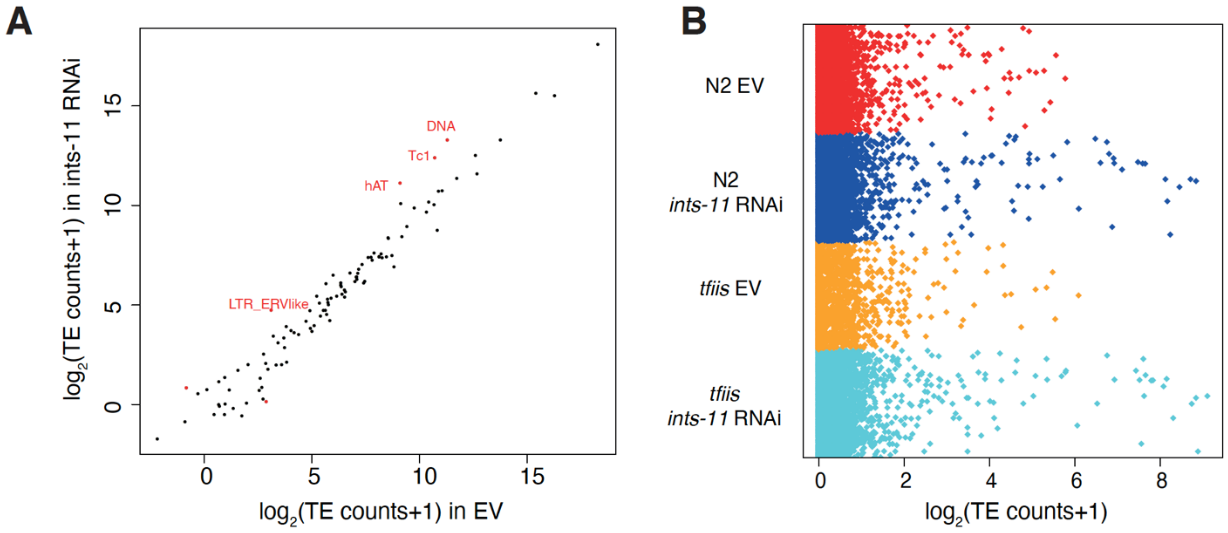

Appendix Figure S7. Transcriptional changes in transposable elements upon ints-11 knockdown.

A. Total CAGE signal summed for all elements from each TE family in EV-treated animals and *int-11* knockdown. TE families showing significant (adjusted p-value<0.05, >2-fold change) are highlighted in red.

B. Dotchart showing the CAGE signal for each individual TE annotated as Tc1 across different conditions. Each point shows the maximum signal across two replicates.

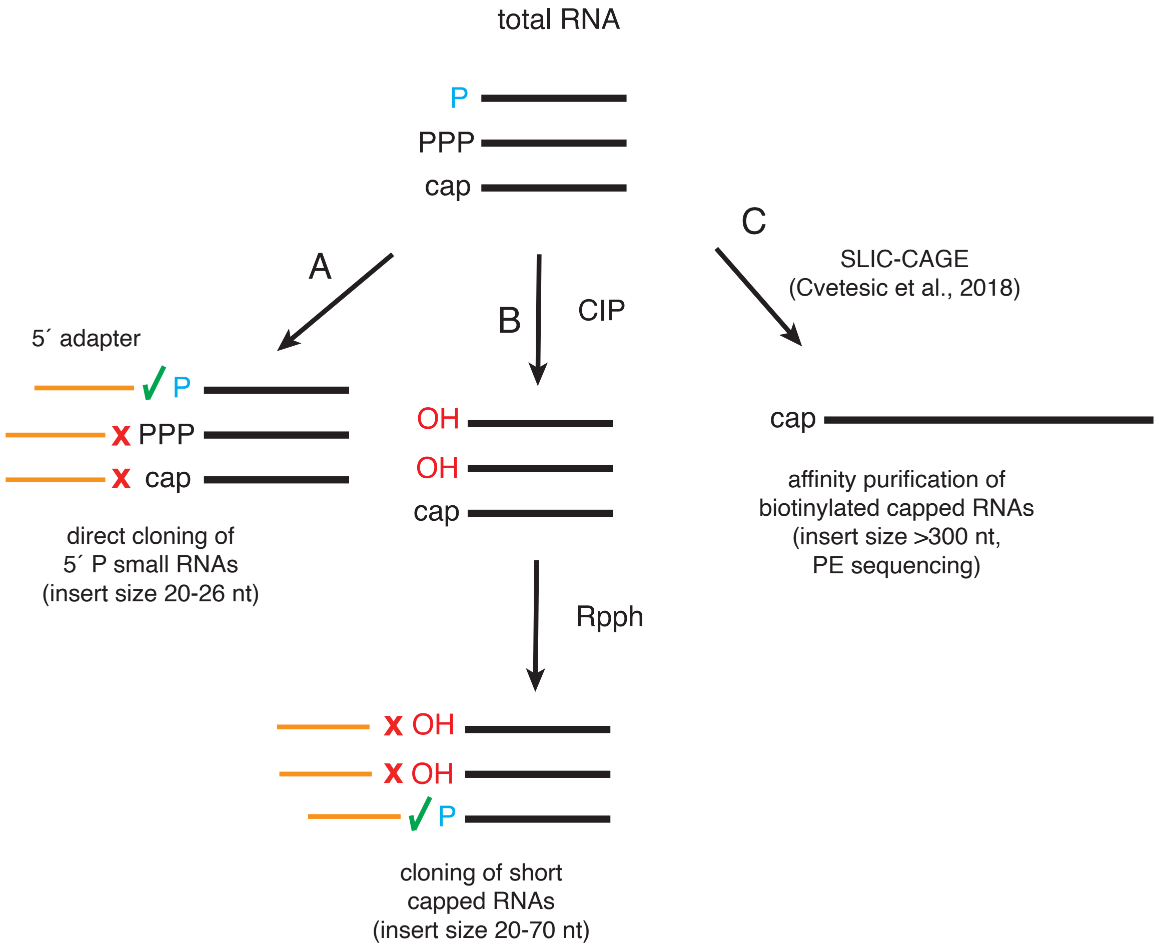

Appendix Figure S8. Overview of cloning methods used in this work.
A. 5´ monophosphate small RNA species (piRNAs, miRNAs) were directly cloned from total RNA samples without enzymatic conversion of 5´ ends.
B. Short capped RNA cloning was carried after sequential treatment of total RNA samples: (1) samples were treated with CIP to dephosphorylate 5´P and 5´PPP species, and (2) the purified resulting RNA was treated with Rpph to convert 5´capped ends into clonable 5´P. This approach enriches capped RNA species in the final library. We purified these libraries at ranges of 18-36 nt or 18-75 nt insert sizes.
C. SLIC-CAGE allows the specific capture of long capped RNAs (>300 nt) via affinity purification of biotin-modified capped 5´ ends.

2. Appendix tables

Appendix Table S1. *C. elegans* strains

Strain	Genotype	Description	Reference
SX1316	mjIs144 II	piRNA sensor strain	(Bagijn et al., 2012)
JCP383	ints-6 (tm1615) IV; jcpSi10 [pJC51 (ints-6p::ints-6::3xFLAG::eGFP::ints-6UTR, unc-119(+))] II	INTS-6::eGFP rescue strain	(Gómez-Orte et al., 2019)
SX2700	mjSi74 I; mjIs144 II; prde-1(mj207)	mCherry::PRDE-1 rescue strain	(Weick et al., 2014)
TEE172	ints-6 (tm1615) IV; jcpSi10 [pJC51 (ints-6p::ints-6::3xFLAG::eGFP::ints-6UTR, unc-119(+))] II; mjSi74 I	Generated by crossing JCP383 and SX2700	This study

Appendix Table S2. Oligonucleotides

Ints-11_Nterm_F	AAAATCCTATTTCGCGAAGGC	Genotype FLAG::ints-11
Ints-11_Nterm_R	CGACCGTCCAACATCTTGTC	Genotype FLAG::ints-11
Ints-11_Nterm_crRNA	CTTGATTTCAGGAATTTTGT	crRNA
FLAG::Ints-11 repair oligo	GTAGAACTTTATAGCTATATTTCA CAATGCCCGACGATTACAAGGA TGACGACGATAAGAAGATACCT GAAATCAAGGTGAGTTCTGAAG GACTTTTCTG	repair template
Ints-6_3UTR_F	ACAACTCACTGAACGTCTGC	Genotype jcpSi10
Ints-6_3UTR_R	ACAAAAGTTTTGAAAGAGCACCT	Genotype jcpSi10
Ints-6_tm1616_F	AGGTGGACGAAGCTTCTCAA	Genotype *tm1616*
Ints-6_tm1616_R	GCATTGGGAAATTGAATGGCA	Genotype *tm1616*
Wormcherry_F	GGAACACAAACCGCAAAACT	Genotype mjSi74 I
Wormcherry_R	GCACCGTCTTCAGGGTACAT	Genotype mjSi74 I
MosSCI Chr I F	GAAAAACCCCGAATTTTGGT	Genotype mjSi74 I
MosSCI Chr I R	GGTGGGAACCTTTTCGTTTT	Genotype mjSi74 I
