# Supplementary material for: Integrator is recruited to promoter‐proximally paused RNA Pol II to generate Caenorhabditis elegans piRNA precursors
Source: EMBO J. 2020 Dec 19;40(5):e105564. doi: 10.15252/embj.2020105564 (PMC7917550; doi:10.15252/embj.2020105564)
Supplement: Supplementary file 2 — Expanded View Figures PDF [file EMBJ-40-e105564-s002.pdf]

Expanded View Figures

Figure EV1. Quantification of total mature piRNA and piRNA precursor levels after RNAi knock-down of *ints*-11 (relates to Fig 1).

A, B Total piRNA read counts normalized using DEseq2 size factors derived from miRNA counts (A) or normalized to the total number of non-structural mapped reads (B). Normalized abundance for each pair of EV and *ints*-11 RNAi samples is shown relative to EV.

C Total piRNA precursor counts normalized to the total number of short-capped RNA reads mapping to WormBase TSSs (Chen *et al*, 2013). Normalized abundance for each pair of EV and *ints*-11 RNAi samples is shown relative to EV.

D Representative examples of a silenced and a desilenced piRNA sensor transgene in the *C. elegans* germline. The proportion of animals desilencing the piRNA sensor upon empty vector and *ints*-11 RNAi treatment is shown in the right panel.

E Precursor length distributions in short-capped RNA libraries (18-36 nt insert size) in EV and *ints-11* RNAi-treated animals.

F Distributions of fold changes in motif-independent piRNA abundance in *ints-11* RNAi-treated animals compared with EV controls.

G Median length increase in precursor length (75 nt insert size libraries) in *ints-11* RNAi-treated animals compared with EV controls.

H Precursor length heat maps outlining the distributions of lengths of unique precursors across piRNA loci in EV controls, and *ints-11* RNAi-treated nematodes.

© 2020 The Authors

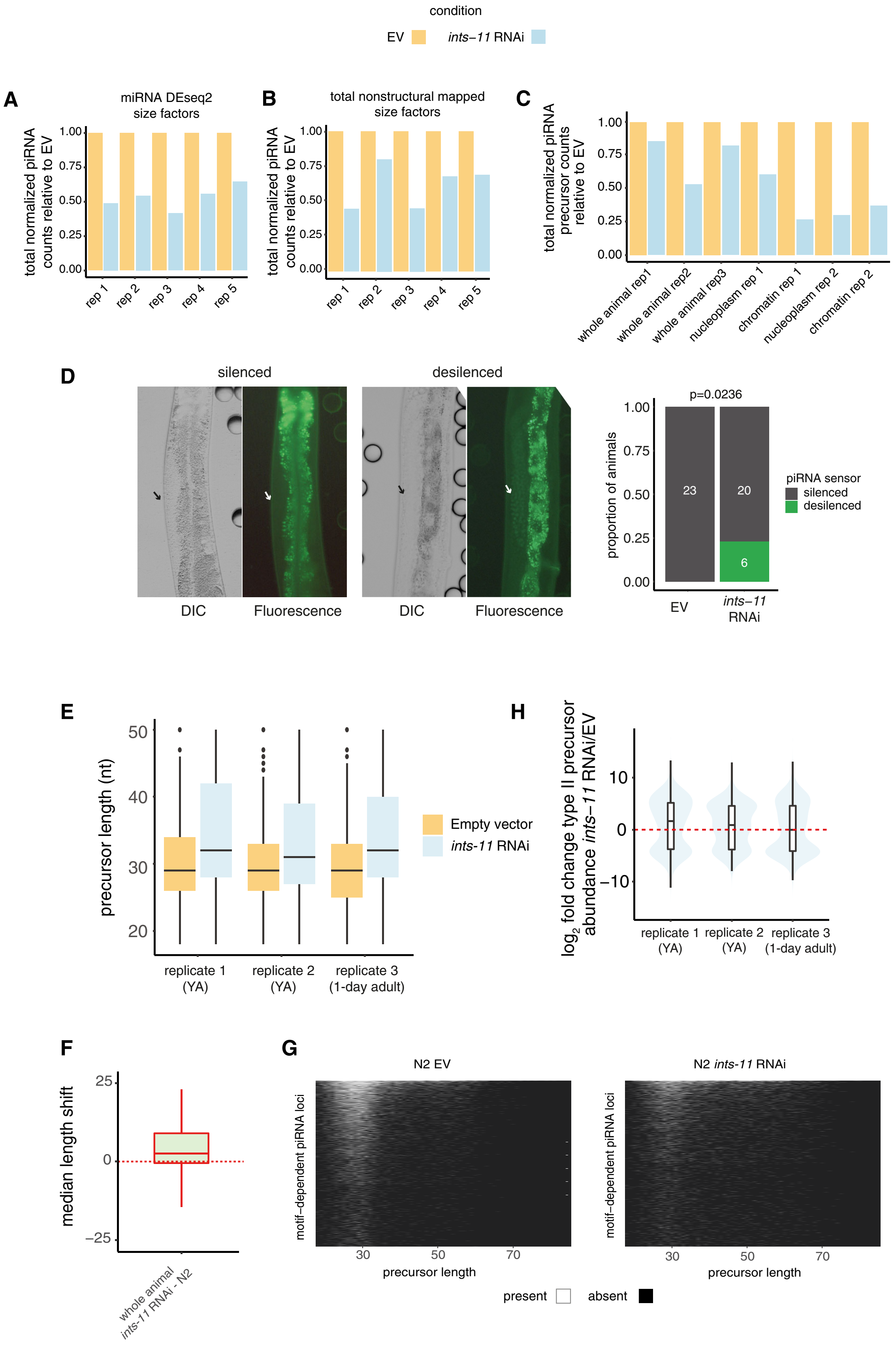
condition
EV
ints−11 RNAi
A
miRNA DEseq2 size factors
total normalized piRNA counts relative to EV
rep 1
rep 2
rep 3
rep 4
rep 5
B
total nonstructural mapped size factors
C
total normalized piRNA precursor counts relative to EV
whole animal rep1
whole animal rep2
whole animal rep3
nucleoplasm rep 1
chromatin rep 1
nucleoplasm rep 2
chromatin rep 2
D
silenced
desilenced
DIC
Fluorescence
p=0.0236
proportion of animals
23
20
6
EV
ints−11 RNAi
piRNA sensor
silenced
desilenced
E
precursor length (nt)
Empty vector
ints-11 RNAi
replicate 1 (YA)
replicate 2 (YA)
replicate 3 (1-day adult)
H
log2 fold change type II precursor abundance ints−11 RNAi/EV
F
median length shift
whole animal ints-11 RNAi - N2
G
N2 EV
N2 ints-11 RNAi
motif−dependent piRNA loci
precursor length
present
absent

Figure EV1.

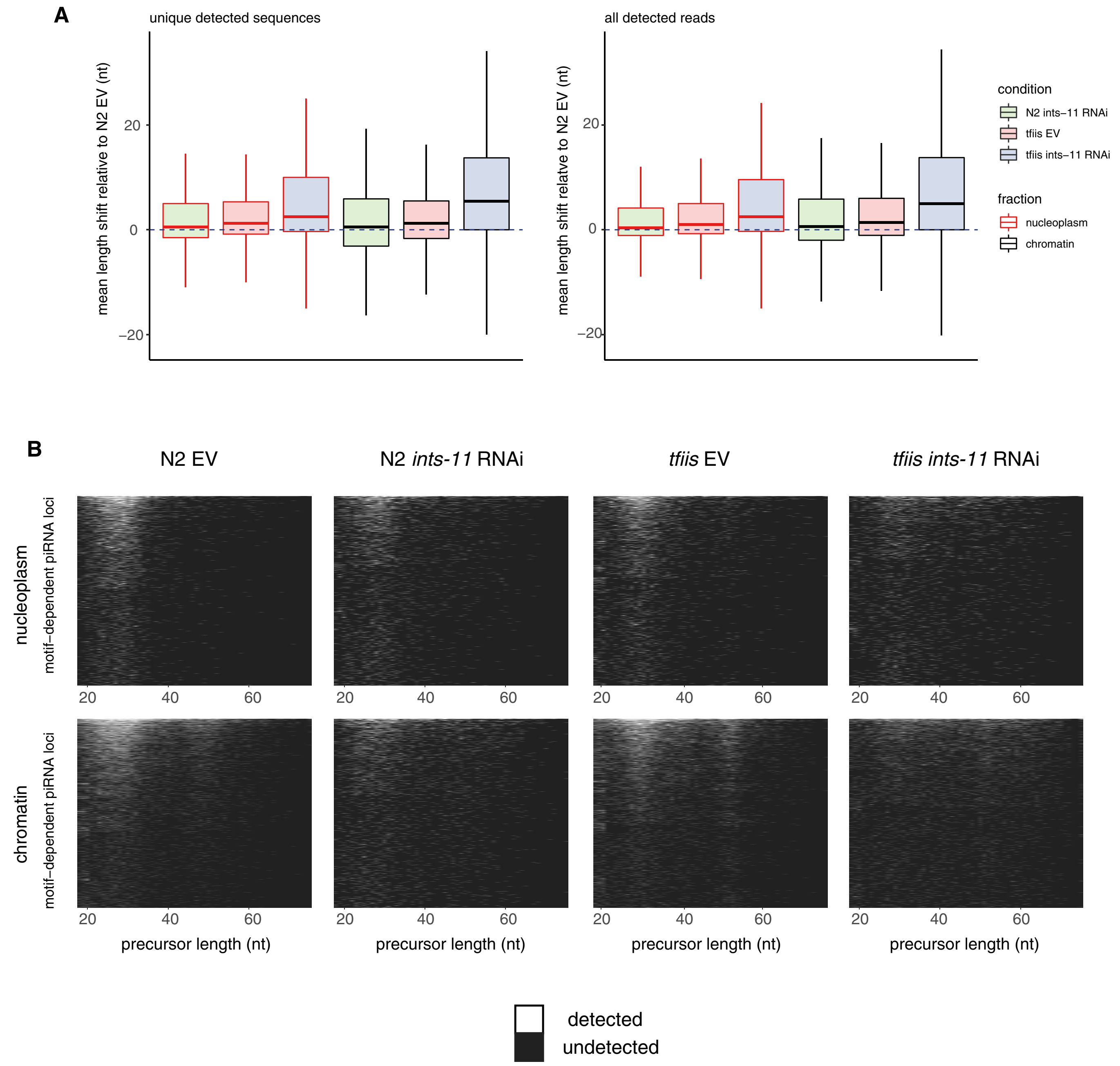

Figure EV2. Locus-by-locus analysis of piRNA precursor length.

A Distributions of mean length changes in precursor length in a locus-by-locus basis (see Materials and Methods). The mean length change (in nt) in each of the genotypes/conditions relative to N2 EV controls was calculated and plotted as a boxplot.

B piRNA precursor length distributions represented as a heat map, where each row corresponds to a piRNA TSS.

 © 2020 The Authors

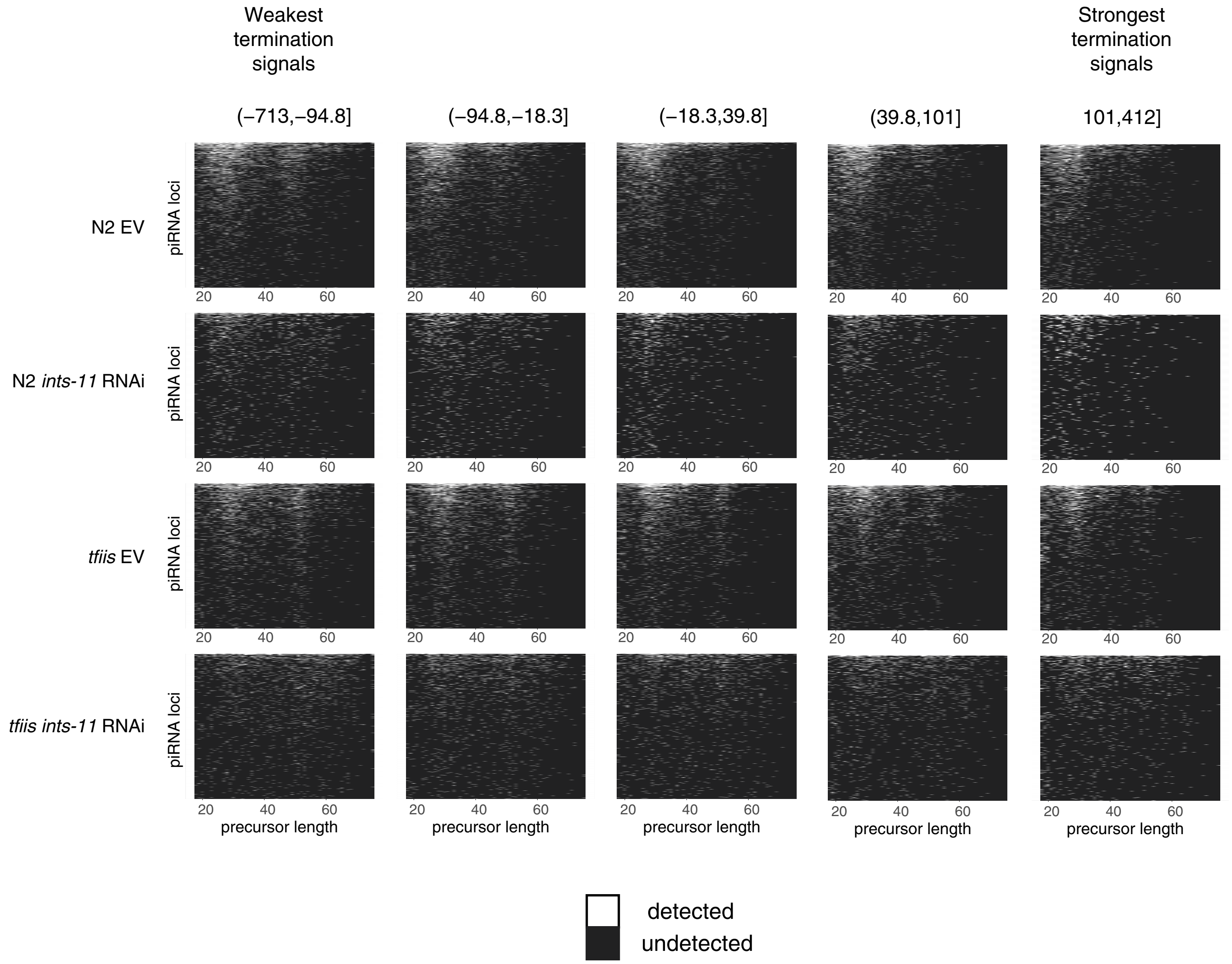

Figure EV3. Analysis of piRNA precursor length across bins of termination signal strength.
Chromatin-bound piRNA precursor length heat maps stratified in bins of increasing termination signal strength.

Figure EV4. Nascent SLIC-CAGE detects readthrough transcription from piRNA loci (relates to Fig 5).

A Clustered correlation matrix of promoter-mapping CAGE tpms for the top 1,000 genes with the highest CAGE signal.
B Intron retention ratio distributions across nascent RNA samples, and in a total RNA control, estimated through IRFinder analysis.
C, D Total raw CAGE tags mapping between −10 and −1 nucleotide positions upstream of motif-dependent piRNAs (C) and motif-independent piRNAs (D).
E Total unique CAGE-detected TSSs mapping between −10 and −1 nucleotide positions upstream of motif-independent piRNAs.
F Mean normalized expression levels of CAGE-detected and non-detected motif-dependent piRNAs at the short-capped RNA level.
G Downsampling analysis of CAGE libraries showing that the detection of piRNA loci is saturated with sequencing depth. Total detected loci in each of the two replicates were averaged for each bootstrapped sample.

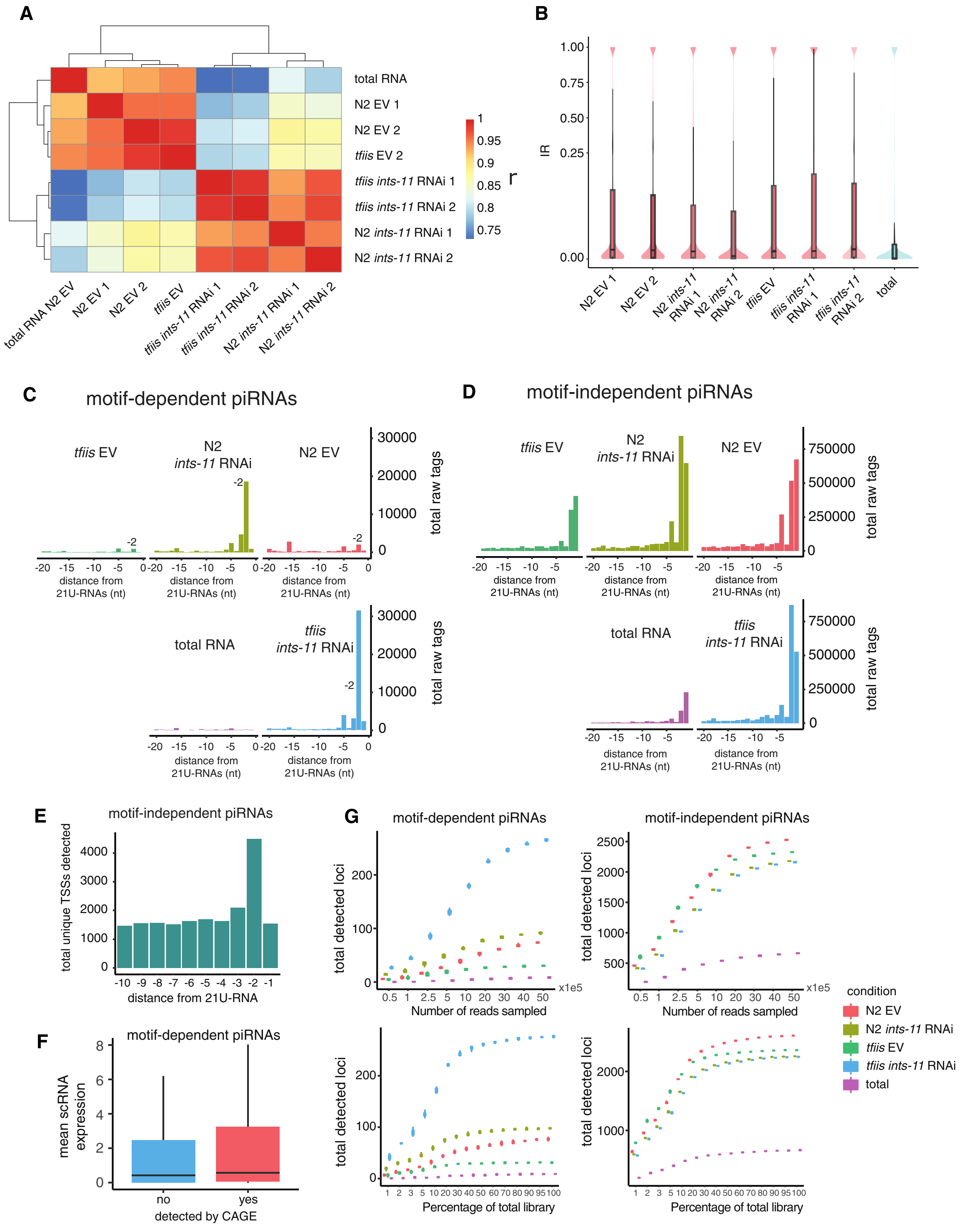

Figure EV4.

 © 2020 The Authors

Figure EV5. Accessibility and H3K27 acetylation levels at piRNA promoters (relates to Fig 5).

A ATAC-seq read coverage around motif-dependent and motif-independent piRNA promoters, in wild-type young adult wild-type worms, and in day 1 adult germlineless glp-1 mutant worms lacking a germline. “Motif-dependent CAGE” refers to motif-dependent loci detected by CAGE.

B Log_2 ratio in accessibility signal at motif-dependent piRNA loci between young adult wild-type worms and day 1 adult germlineless glp-1 mutants lacking a germline. This reflects a specific increase in accessibility at motif-dependent loci in germ cells. “Motif-dependent CAGE” refers to motif-dependent loci detected by CAGE.

C H3K27ac ChIP signal minus input around motif-dependent and motif-independent piRNA loci in isolated germ nuclei (left panel), and in isolated somatic nuclei (right panel).

D, E Distributions of H3K27ac (D) and accessibility signal (E) in motif-dependent and motif-independent piRNA loci.

© 2020 The Authors

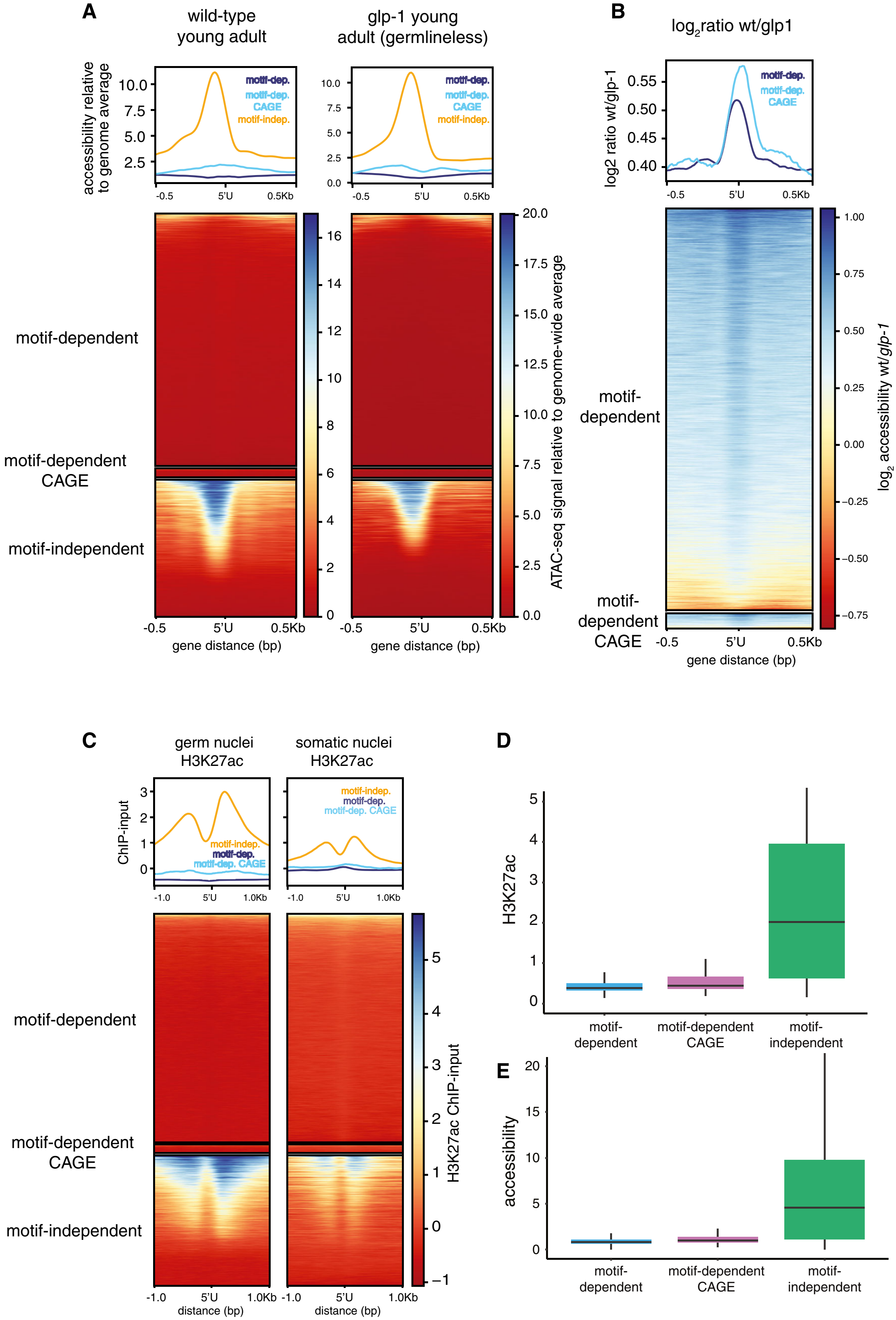

Figure EV5.

© 2020 The Authors
